# Dyslipidemia among Patients with Type 2 Diabetes Mellitus Visiting a Tertiary Care Centre

**DOI:** 10.31729/jnma.8306

**Published:** 2023-10-31

**Authors:** Bikram Khadka, Sundar Pandey, Deepak Kafle

**Affiliations:** 1 Department of Biochemistry, Devdaha Medical College and Research Institute, Devdaha, Rupandehi, Nepal; 2 Department of Internal Medicine, Devdaha Medicaf College and Research Institute, Devdaha, Rupandehi, Nepal; 3 Department of Biochemistry, Chitwan Medical College Teaching Hospital, Bharatpur, Chitwan, Nepal

**Keywords:** *cardiovascular disease*, *dyslipidemia*, *prevalence*, *type 2 diabetes mellitus*

## Abstract

**Introduction:**

A triad of lipid and lipoprotein metabolism is known as dyslipidemia. Dyslipidemia is one of the major risk factors for cardiovascular diseases in diabetes mellitus which is a leading cause of morbidity and mortality worldwide. The aim of the study was to find out the prevalence of dyslipidemia among patients with type 2 diabetes mellitus visiting a tertiary care centre.

**Methods:**

A descriptive cross-sectional study was conducted in a tertiary care centre among patients with type 2 diabetes mellitus from 18 February 2020 to 18 August 2020 after obtaining ethical clearance from the Institutional Review Committee. Demographic and blood samples were analysed and recorded using validated and calibrated tools. A convenience sampling technique was used. The point estimate was calculated at a 95% Confidence Interval.

**Results:**

Out of 390 patients with type 2 diabetes mellitus, 343 (87.95%) (84.72-91.18, 95% Confidence Interval) had dyslipidemia. The most prevalent dyslipidemia was high low-density lipoprotein cholesterol at 85 (24.78%) followed by mixed dyslipidemia at 305 (88.92%).

**Conclusions:**

The prevalence of dyslipidemia among patients with type 2 diabetes mellitus was found to be higher than studies conducted in similar settings. We recommend regular testing of blood glucose and blood lipid levels for early detection of dyslipidemia and putting them under medical supervision to reduce the unwanted complications of cardiovascular diseases.

## INTRODUCTION

Dyslipidemia refers to a triad of abnormal lipid and lipoprotein metabolism.^1^ Dyslipidemia among type 2 diabetes mellitus (T2DM) is an important modifiable risk factor for atherosclerosis, stroke and cardiovascular diseases (CVD).^2^ It is mainly due to increased free fatty acids flux secondary to insulin resistance.^3^

Studies from Nepal reported a prevalence of 61.0 to 90.7% of dyslipidemia among T2DM from different communities.^4^ Given that people with T2DM are strongly associated with atherogenic dyslipidemia. Atherogenic dyslipidemia in T2DM shortens life expectancy and multiplies the risk of CVD.^5^ Early detection and aggressive management of dyslipidemia in T2DM patients decreases the likelihood of major comorbidities resulting from such lipid abnormalities, such as CVD.^6^

The aim of the study was to find out the prevalence of dyslipidemia among patients with type 2 diabetes mellitus visiting a tertiary care centre.

## METHODS

A descriptive cross-sectional study was conducted at Devdaha Medical College and Research Institute, Devdaha, Rupandehi, Nepal from 18 February 2020 to 18 August 2020. Ethical clearance was obtained from the Institutional Review Committee (Protocol number: 004/020). Patients with T2DM with an age of more than 30 years who attended the Outpatient Department of the institute were enrolled after obtaining written consent.

The patients with gestational diabetes, diabetic ketoacidosis, thyroid disorder, renal failure, hepatic diseases, acute illnesses, recurrent myocardial infarction, unstable angina and drug therapy that interfered with the serum lipid levels and those denied written consent were excluded from the study. Convenience sampling was used. The sample size of the study was calculated using the formula:


$$\begin{array}{ll} \text{n} & ={\text{Z}}^{2}\times \frac{\text{p}\times \text{q}}{{\text{e}}^{\text{2}}}\\ & =1.96^{2}\times \frac{0.50\times 0.50}{0.05^{2}}\\ & =384 \end{array}$$

Where,

n = minimum required sample size
Z = 1.96 at 95% Confidence Interval (CI)
p = prevalence taken as 50% for maximum sample calculation
q = 1-p
e = margin of error, 5%

The calculated sample size was 384. However, we took 390 samples.

Personal profiles of study participants like age, sex, duration of T2DM, and smoking habits were obtained through comprehensive baseline questionnaire. Smoking habit was noted as yes if the participant smoked at least one cigarette per day. Height in centimeter (cm) and weight in kilogram (kg) was measured by using a stadiometer and portable weighing machine respectively without footwear and under light clothing. Body mass index (BMI) of each individual was calculated as their weight (kg) divided by the square of their height (m^2^). Obesity was defined according to Asia Pacific guidelines for South Asia. According to this, individuals with BMI between 23 and 25 kg/m^2^ were defined as overweight and obese if their BMI was ≥25 kg/m^2^.^7^

Five milliliter of blood samples were collected under aseptic condition in the morning after an overnight fasting (8 to 12 hours). Collected blood sample was transferred into two different containers, Ethylene Diamine Tetra Acetic Acid (EDTA) for HbA1c and rest in a gel tube. Blood samples within gel tubes were allowed to clot and serum was separated by centrifugation at 3000 RPM for 10 minutes. Separated serum sample was analysed for serum total cholesterol, triglycerides, high-density lipoprotein (HDL) and low-density lipoprotein (LDL) cholesterol by a fully auto Biochemistry analyzer (Agappe CCXL) where as HbA1c was analysed by specific protein analyzer using nephelometry method from Gold site diagnostics.

Diagnosis of T2DM was done according to American Diabetes Association (ADA) 2016 criteria. Poor diabetic control was defined as HbA1c >7%.^8^ National Cholesterol Education Programme (NCEP) Adult Treatment Panel III (ATP III) guideline was adopted for reference level of serum lipid and definition of dyslipidemia.^9^ Single dyslipidemia was defined as the existence of only one abnormal lipid parameter of the individual mentioned before whereas mixed or combined dyslipidemia was defined as the combination of two or more dyslipidemia.

Data were entered into Microsoft Excel 2010 and analysis was performed using IBM SPSS Statistics version 20.0. The point estimate was calculated at a 95% Confidence Interval.

## RESULTS

Among 390 patients with type 2 diabetes mellitus, 343 (87.95%) (84.72-91.18, 95% CI) had dyslipidemia. The most prevalent dyslipidemia was high LDL cholesterol at 85 (24.78%) followed by mixed dyslipidemia in 305 (88.92%). Likewise, dyslipidemia was more prevalent in males 192 (55.98%) (Table 1).

**Table 1:** Abnormal lipid profile among T2DM patients (n = 343).

Parameters		n (%)
**Gender**	Male	192 (55.98)
	Female	133 (38.77)
	High TC	190 (55.39)
**Lipid profile**	Isolated high TC	1 (0.29)
	High TG	246 (71.72)
	Low-HDL	251 (73.18)
	Isolated low-HDL	7 (2.04)
	High LDL	278 (81.05)
	Isolated high LDL	85 (24.78)
	Mixed dyslipidemis	305 (88.92)

Among 343 patients, 169 (49.27%) were in the age group of 45-59 years (Table 2).

**Table 2:** Various characteristics of diabetic patients (n= 343).

Parameters	n (%)
**Age group (years)**	
30-44	160 (46.65)
45-59	169 (49.27)
>60	14 (4.08)
**BMI**	
≤25	61 (17.78)
≥25	282 (82.21)
Smoking habit	129 (37.70)
**Glycemic control**	
HbA1c<7	104 (30.32)
HbA1c≥7	239 (69.70)

## DISCUSSION

Among 390 patients with type 2 diabetes mellitus, 343 (87.95%) had dyslipidemia. This finding of our study accords with the study performed in Kaski, Nepal.^4^ However, several researches from other areas of Nepal have shown different prevalence and patterns of dyslipidemia.^10^ Such variation in the prevalence and pattern of dyslipidemia among diabetes participants may be due to variation in ethnic groups of people living in a different part of Nepal and each ethnic group have variation in socioeconomic status, lifestyle, dietary habit and cultural practices. Our study demonstrated a higher prevalence of dyslipidemia among males 89.3% than females 76.6%. This finding of our study is comparable to a similar study done in Nepal.^11^ However many previous studies reported no significant difference between lipid parameters among male and female participants.^4,12^

We next analysed the prevalence of subtypes of dyslipidemia. The most prevalent subtype of dyslipidemia was high LDL cholesterol (81.0%), followed by mixed dyslipidemia (78.2%) Low HDL cholesterol (73.17%), hypertriglyceridemia (71.7%) and high total cholesterol (38.7%). Our findings are in agreement with previous reports.^4,11^

According to Diabetes Complications and Control Trial (DCCT), HbA1c has been established as a gold standard parameter to monitor glycemic control. HbA1c value <7% is determined to be appropriate for reducing the risk of cardiovascular complications. As expected we found a higher prevalence of High Total cholesterol, hypertriglyceridemia, low HDL and mixed dyslipidemia among the patients with poor glycemic control. This finding of our study is consistent with the findings of many other studies conducted among T2DM patients.^13,14^

Similarly, our findings revealed that dyslipidemia was more prevalent in T2DM patients having higher BMI. Mixed dyslipidemia (78.5%) was most prevalent followed by high LDL cholesterol. (65.9%) Several cross-sectional studies conducted in Nepal and elsewhere have also shown similar results.^11,15^ The increased rate of lipolysis in adipocytes and increased input of free fatty acids into the liver due to insulin resistance in T2DM and obesity increases the production of triglyceride-rich lipoprotein. In addition, due to the decreased activity of endothelial-bound enzyme lipoprotein lipase, there is delayed clearance of such lipoproteins leading to dyslipide mia.^16^

Although the study has met its aim, there were some unavoidable circumstances. First, our study included only hospital-based samples which may not truly represent the diabetic population of this region. The information which affects the blood lipid level like caloric intake, diet (vegetarian or non-vegetarian), place of residency (urban or village), ethnic group, socio-economic status, cultural practices, and lifestyle were not assessed in this study. Therefore the findings of this study should be interpreted within a limited context and cannot be generalized to the whole diabetic population.

## CONCLUSIONS

The prevalence of dyslipidemia among patients with type 2 diabetes mellitus was found to be higher than other studies conducted in similar settings. We recommend regular testing of blood glucose and blood lipid levels for early detection of dyslipidemia and putting them under medical supervision to reduce the unwanted complications of cardiovascular diseases. Our study suggests health policy planners formulate and implement policies that aim to increase public awareness about diabetic dyslipidemia, healthy diet and aggressive lifestyle changes such as physical exercise and weight reduction and effective medication to obtain glycemic control and normal lipid levels. Further, studies covering the population of the whole nation to support the generalizability of the study are needed.
